# Pelvic organ movements in asymptomatic nulliparous and symptomatic premenopausal women with pelvic organ prolapse in dynamic MRI: a feasibility study comparing midsagittal single-slice with multi-slice sequences

**DOI:** 10.1007/s00261-023-03944-8

**Published:** 2023-05-19

**Authors:** Soleen Ghafoor, Stephan M. Beintner-Skawran, Gian Stöckli, Cornelia Betschart, Cäcilia S. Reiner

**Affiliations:** 1 Institute of Diagnostic and Interventional Radiology, University Hospital Zurich, Raemistrasse 100, 8091 Zurich, Switzerland; 2 Department of Nuclear Medicine, University Hospital Zurich, Raemistrasse 100, 8091 Zurich, Switzerland; 3 Department of Gynecology, University Hospital Zurich, Frauenklinikstrasse 10, 8091 Zurich, Switzerland; 4 University of Zurich, Zurich, Switzerland

**Keywords:** Pelvic organ prolapse, Magnetic resonance imaging, Pelvic floor, Pelvic floor disorders

## Abstract

**Purpose:**

To compare multi-slice (MS) MRI sequences of the pelvis acquired at rest and straining to dynamic midsagittal single-slice (SS) sequences for the assessment of pelvic organ prolapse (POP).

**Methods:**

This IRB-approved prospective single-center feasibility study included 23 premenopausal symptomatic patients with POP and 22 asymptomatic nulliparous volunteers. MRI of the pelvis at rest and straining was performed with midsagittal SS and MS sequences. Straining effort, visibility of organs and POP grade were scored on both. Organ points (bladder, cervix, anorectum) were measured. Differences between SS and MS sequences were compared with Wilcoxon test.

**Results:**

Straining effort was good in 84.4% on SS and in 64.4% on MS sequences (*p* = 0.003). Organ points were always visible on MS sequences, whereas the cervix was not fully visible in 31.1–33.3% on SS sequences. At rest, there were no statistically significant differences of organ point measurements between SS and MS sequences in symptomatic patients. At straining, positions of bladder, cervix, and anorectum were + 1.1 cm (± 1.8 cm), − 0.7 cm (± 2.9 cm), and + 0.7 cm (± 1.3 cm) on SS and + 0.4 mm (± 1.7 cm), − 1.4 cm (± 2.6 cm), and + 0.4 cm (± 1.3 cm) on MS sequences (*p* < 0.05). Only 2 cases of higher-grade POP were missed on MS sequences (both with poor straining effort).

**Conclusion:**

MS sequences increase the visibility of organ points compared to SS sequences. Dynamic MS sequences can depict POP if images are acquired with sufficient straining effort. Further work is needed to optimize the depiction of the maximum straining effort with MS sequences.

**Graphical abstract:**

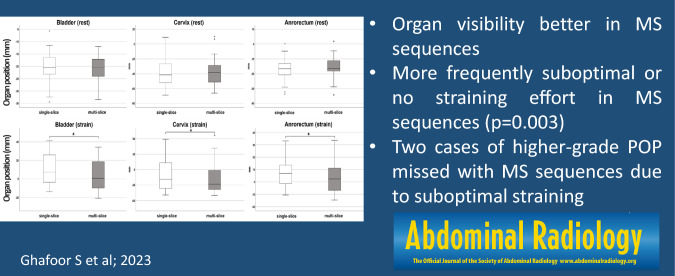

**Supplementary Information:**

The online version contains supplementary material available at 10.1007/s00261-023-03944-8.

## Introduction

Female pelvic floor dysfunction is a condition characterized by weakened supporting structures and presents as pelvic organ prolapse (POP), incontinence, and sexual dysfunction. It is a prevalent condition in women, affecting 30–50% of the general population [1]. The prevalence increases with parity and age. POP is characterized by descent of pelvic organs from their normal position usually in a gravity-dependent manner along the longitudinal body axis causing a multitude of symptoms depending on the severity and compartmental involvement. The condition has significant implications on the quality of life and physical well-being of affected women [2].

The female pelvic floor is a sophisticated three-dimensional (3D) unit that is anatomically partitioned into three compartments: the anterior, middle, and posterior compartments. The anterior compartment comprises the bladder and urethra, the middle compartment encompasses the uterus, cervix, and vagina, while the posterior compartment houses the rectum, anal canal, and anal sphincter.

Magnetic resonance imaging (MRI) can aid in diagnosing POP and planning of treatment strategy by providing detailed information about pelvic floor anatomy and structural morphology. Both static and dynamic MRI using straining maneuvers enable a comprehensive interpretation of the complex pelvic floor anatomy at rest and during straining [3–5].

On dynamic MR images, the degree of descent of the different pelvic compartments is assessed through measuring differences in organ position between images at rest and during straining in relation to a pelvic reference line [6].

Various pelvic reference lines are available for the measurement of prolapse on MRI [7], but in clinical practice, one of the most commonly used reference line is the “pubococcygeal line” (PCL). The PCL is defined as a line that extends from the most inferior point of the symphysis pubis to the tangent of the last coccygeal joint [8]. Recently, a new reference line system, the pelvic inclination correction system (PICS), which is adjusted for changes in pelvic inclination, has been proposed. In the PICS, a line is drawn from the inferior border of the symphysis pubis to the anterior border of the last sacrococcygeal joint (so called “SCIPP line”) and an additional line is drawn that is rotated 34° (at rest) or 29° (during straining) clockwise to the SCIPP line [6]. This 2-dimensional (2D) PICS system was later enhanced to a “3D PICS” system where any point in the pelvis can be annotated at rest and straining relative to the PICS plane and located within a 3-dimensional (3D) coordinate system [8].

While this newly proposed “3D PICS” system would now allow for organ point measurements in the 3D space, dynamic pelvic floor MRI is traditionally performed in a 2D single-slice midsagittal plane [9].

These single-slice sequences do not allow for a dynamic assessment of the pelvic floor in its entirety, as its components represent a complex 3D organizational unit of bones, muscles, ligaments, and fasciae that are not all located in the midline. Therefore, traditional measures of POP based on midsagittal images alone may not accurately reflect the complex 3D structure of the pelvic floor. Furthermore, the pelvic organs are not always strictly aligned in the midsagittal plane; hence their delineation may be challenging in this plane.

Multi-slice sequences acquired at rest and during a straining maneuver could hence improve the dynamic visualization of the critical organ points and pelvic floor structures, which are not all necessarily located in the midsagittal plane and thus allow for measurements in the 3D space. To date, it has not been investigated whether such dynamic multi-slice MR images obtained at rest and straining are comparable to the reference standard of dynamic 2D single-slice images in the midsagittal plane with regards to the diagnosis of pelvic organ descent.

Therefore, the purpose of this prospective feasibility study was to compare multi-slice sequences of the pelvis acquired at rest and straining to the traditional method using conventional dynamic single-slice dynamic sequences in the midsagittal plane for the assessment of pelvic organ descent.

## Materials and methods

### Patients and volunteers

The local ethics committee approved this prospective MRI study (Study-ID: BASEC 2018-01107). All patients and volunteers gave written informed consent.

Patients were included, if they had symptoms of POP as assessed through a standardized pelvic floor questionnaire [10, 11]. Briefly, this is a standardized and validated pelvic floor questionnaire integrating four domains of pelvic floor dysfunction (bladder function, bowel function, sexual function, and pelvic organ prolapse), grades their severity, and assesses bothersomeness and condition-specific quality of life. For each of the four domains, a total value is returned and calculated into a total “pelvic floor dysfunction score” which can reach a maximum of 40 points. The higher the score, the more severe the pelvic floor dysfunction [11]. Furthermore, the degree of POP was assessed through a dedicated clinical urogynecologic examination using the “Pelvic Organ Prolapse Quantification (POP-Q)” system with grades 0 (no prolapse) to 4 (maximum descent) scored according to the extent of organ prolapse relative to the hymen as the anatomic reference point [12, 13].

Asymptomatic volunteers were included if symptoms of pelvic organ prolapse were absent (assessed through a structured interview prior to inclusion) and if they had never given birth (nulliparity). The asymptomatic volunteers were also asked to fill out the standardized questionnaire [11]. The volunteers did not undergo a POP-Q exam.

Inclusion criteria were a given written informed consent, a POP-Q grade of 2 or more in any compartment (for symptomatic patients only), and a complete MRI examination. Exclusion criteria for symptomatic patients or asymptomatic volunteers were inability to comply with instructions during the MRI exam, history of prior pelvic floor surgery and general contraindications to MRI (e.g., presence of non-MR-compatible metallic implants, devices or metallic foreign bodies), incomplete MRI exams, failure to return the standardized questionnaires and failure to undergo the urogynecologic examination for the assessment of the POP-Q grade (for the symptomatic patients).

### MRI protocol

MRI examinations were performed on a 3.0 T clinical MRI scanner (Skyra, Siemens Healthineers, Erlangen, Germany) with a 60-channel array coil. Subjects were examined in supine body position. All women emptied their bladder 15 min prior to the examination and were instructed on how to correctly perform the straining maneuver for the dynamic phases of the examination. For maximum straining, the subjects were instructed to bear down as much as they could, as though they were trying to defecate. Before image acquisition, participants were asked to perform one straining maneuver for practice and performed a “training session” of straining before the single-slice and multi-slice sequences were acquired. Then, the multi-slice sequences were acquired first followed by the single-slice sequences. For multi-slice dynamic imaging, a proton-density-weighted (PDw) sequence was acquired in the axial, sagittal and coronal plane (TSE, TR/TE, 2290/9 ms; voxel size, 0.75 × 0.75 × 6 mm^3^; matrix, 320 × 320; FOV, 240 × 240 mm; slice thickness, 6 mm; acquisition time, 20 s per plane; slice number, 16) at rest and maximum straining maneuver. For the straining maneuver, patients were instructed to perform an increasing straining maneuver and hold the position at maximum straining during image acquisition.

For single-slice dynamic imaging, a single-slice steady-state coherent sequence (true fast imaging with steady state free precession [TRUFI]) was acquired in the mid-sagittal plane (TR/TE, 460/1.5 ms; matrix, 320 × 320; FOV, 240 × 240 mm; slice thickness, 10 mm) at rest and during three consecutive straining maneuvers (total acquisition time 1 min 10 s, 72 consecutive images per straining maneuver). The images from this sequence were then viewed in cine loop mode.

### Image analysis

#### Qualitative image analysis

Straining effort and visibility of the organ points were assessed qualitatively by an abdominal radiologist (S.G., 7 years of experience in pelvic MRI). The straining effort on the single-slice and multi-slice MR images in sagittal plane were assessed subjectively using a 3-point scale (good straining effort, suboptimal straining effort, no or nearly no straining effort). Criteria for a good straining effort were clear movement of the abdominal wall with outward bowing, “swirling” of the bladder content und visible downward movement of the pelvic floor. Furthermore, the visibility of organ points on the single-slice and multi-slice sequence was assessed on the rest and straining images in sagittal plane using a 3-point scale (not visible, partly visible, completely visible).

#### Quantitative analysis

The MR images were loaded onto and annotated with an in-house developed software tool called “3D PICS” (hereinafter referred to as “PICS tool”) [8]. Details on this software tool have been described elsewhere [8]. Briefly, this tool is based on a 3D coordinate system using pre-defined bony landmarks (the inferior margin of the symphysis, the sacrococcygeal joint, and the ischial spines). Based on this pre-defined coordinate system using these easily identifiable bony landmarks, the *x*- (antero-posterior location), *y*- (cranio-caudal location), and *z*-coordinates (medio-lateral location) of any given annotated structure on the MR images can be assessed within this 3D grid at rest and straining. The “3D PICS” tool uses the PICS plane as a standardized reference plane that accounts for the changes in pelvic inclination in supine position and for differences in inclination between rest and straining maneuvers. The rationale for the use of the PICS plane is based on previous research [6, 14]. For this study, organ point locations of the three pelvic compartments, namely the bladder base for the anterior compartment, the external cervical os for the middle compartment, and the anorectal junction for the posterior compartment were annotated on the single-slice and multi-slice sequences at rest and at straining in sagittal plane.

All annotations were made by an abdominal radiologist (S.G., 7 years of experience in pelvic MRI) using the PICS tool. For assessment of interreader agreement, a random subset of 20 cases (patients, *n* = 10; asymptomatic controls, *n* = 10) was also evaluated by a second reader (G.S., gynecology resident, basic experience in pelvic MRI).

The coordinates from these annotations were exported for further analysis. Coordinates of organ points above the PICS plane have a negative sign while those located below the PICS plane have a positive sign.

### Grading of POP

For the grading of POP, the PCL was used as reference line, as there are established cut-off values for this reference line. Images were reviewed on a commercial picture archiving and communication system (PACS) workstation. The same pelvic organ points in relation to the PCL at rest and straining were annotated on the single-slice and multi-slice sequences by an abdominal radiologist (S.G.) to grade the pelvic organ descent according to published consensus recommendations [15] (Supplementary Table 1). There was a 14-day interval between measurements on the single-slice and multi-slice sequences to reduce recall bias. The POP grades based on measurements in the single-slice sequence served as the reference standard as this is the currently established method [15].

### Statistical analysis

Demographics of the study population and readout results were analyzed using descriptive statistics. Categorical variables were described as frequencies and percentages. Continuous variables were described as means and standard deviations or medians and interquartile range.

Differences in the straining effort between the two sequence types were analyzed with the Wilcoxon signed rank test. Differences in the visibility of the organ points on the single-slice sequence between rest and straining images were analyzed with descriptive statistics.

Differences of organ point measurements at rest and straining between single-slice and multi-slice sequences were compared with a Wilcoxon signed rank rest.

Differences between measurements on the two sequence types were analyzed with a Bland–Altman plot (difference plot) [16].

Interobserver agreement for the organ point measurements was analyzed with the intraclass correlation coefficient (ICC). ICCs were interpreted as follows: < 0.40, poor agreement; 0.40–0.59, fair agreement; 0.60–0.74, good agreement; and ≥ 0.75, excellent agreement [17].

The data was tested for normal distribution using the Kolmogorov–Smirnov test. A 2-tailed *p*-value of < 0.05 was used to determine the statistical significance. Statistical analyses were performed with SPSS (version 22, IBM Corporation, Armonk, NY, USA).

## Results

### Patient and volunteer characteristics

Two symptomatic patients were excluded (unable to follow instructions during the MRI exam and incomplete exam *n* = 1, failure to return the questionnaire and undergo urogynecologic examination for POP-Q grading *n* = 1). The final population comprised 23 consecutive premenopausal patients with symptomatic POP and 22 asymptomatic premenopausal nulliparous volunteers. Baseline characteristics of the patients and volunteers are depicted in Table 1.

**Table 1:** Baseline characteristics of patients and healthy volunteers

	Patients (*n* = 23)	Volunteers (*n* = 22)
Age (years)	39.2 ± 4.5	24.1 ± 3.7
BMI (kg/m^2^)	22.3 ± 3.8	21.9 ± 3.1
Parity	2 (1–3)	–
Clinical POP-Q		
Anterior compartment		
Grade 0	0	
Grade 1	7 (30.4%)	
Grade 2	14 (60.9%)	
Grade 3	2 (8.7%)	
Grade 4	0	
Middle compartment		
Grade 0	4 (17.4%)	
Grade 1	14 (60.9%)	
Grade 2	3 (13.0%)	
Grade 3	2 (8.7%)	
Grade 4	0	
Posterior compartment		
Grade 0	5 (21.8%)	
Grade 1	9 (39.1%)	
Grade 2	6 (26.1%)	
Grade 3	3 (13.0%)	
Grade 4	0	
Pelvic floor dysfunction score*	8.7 (6.8–11.3)	1.6 (0.3–2.6)

Data is presented as either mean ± standard deviation, numbers (percentage), or median (range or interquartile range)

*BMI* body mass index, *POP-Q* Pelvic Organ Prolapse Quantification System

*Assessed through analysis of the standardized questionnaire

Differences in age and pelvic dysfunction scores (obtained through pelvic floor questionnaires) between symptomatic patients and asymptomatic volunteers were statistically significant (*p* < 0.001). Differences in body mass index were not statistically significant (*p* = 0.895).

### Qualitative image analysis

#### Straining effort

**Fig. 1:**
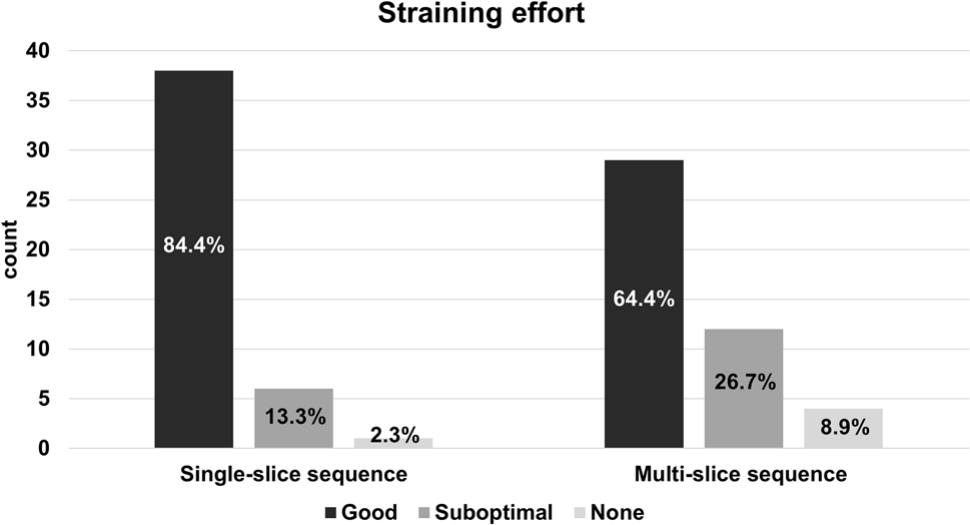
Bar chart depicting the differences in distribution and proportion of subjectively assessed straining effort in the single-slice and multi-slice sequences (*p* = 0.003)

On the single-slice sequences, straining effort was good in 38 cases (84.4%), suboptimal in 6 cases (13.3%) and no straining effort was seen in one case (2.3%). On the multi-slice sequences, straining effort was good in 29 cases (64.4%), suboptimal in 12 cases (26.7%), and absent in 4 cases (8.9%). There was a statistically significant difference between the single-slice and multi-slice sequence (*p* = 0.003) (Fig. 1).

#### Visibility of organ points

The organ points were always visible in the multi-slice sequence. For the single-slice sequence, the bladder point was seen in all cases (100%) at rest and during straining. At rest, the cervix point was not visible in 5 cases (11.1%), partly visible in 9 cases (20%) and fully visible in 31 cases (68.9%). At straining, the cervix point was not visible in 6 cases (13.3%), partly visible in 9 cases (20%) and fully visible in 30 cases (66.7%). At rest and during straining, the anorectal junction was partly visible in one case (2.2%) and completely visible in all other cases (97.8%) (Supplementary Fig. 1).

### Quantitative analysis

#### Pelvic organ point measurements in asymptomatic volunteers

**Fig. 2:**
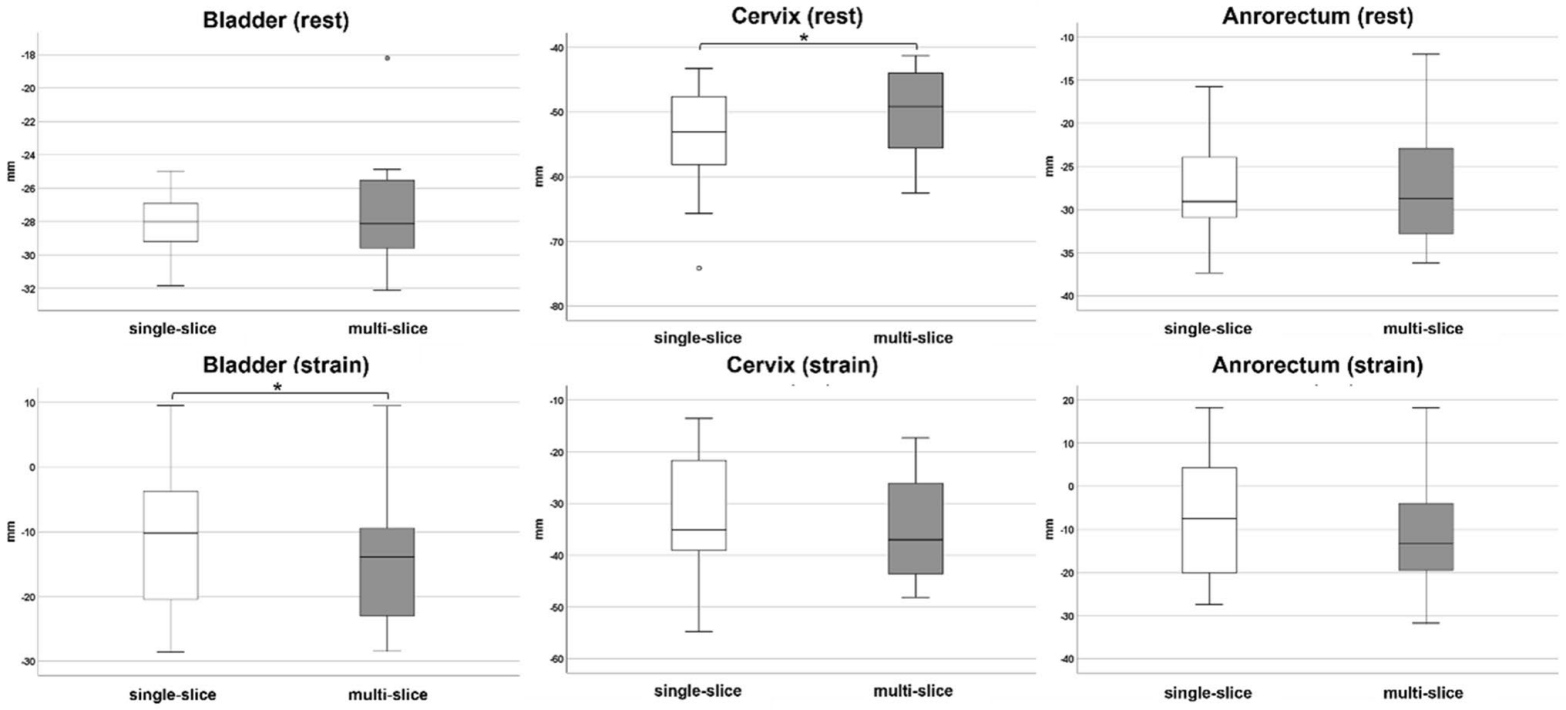
Box plots comparing organ positions in the *y*-axis at rest (top row) and straining (bottom row) between single-slice (white box) and multi-slice sequences (grey box) in the asymptomatic volunteers. Asterisks indicate statistical significance

At rest, the mean positions of bladder, cervix, and anorectal junction were − 2.8 cm (± 0.2 cm), − 5.4 cm (± 0.8 cm), and − 2.8 cm (± 0.6 cm) in the single-slice sequences. Same organ positions were − 2.7 cm (± 0.3 cm), − 5.0 cm (± 0.7 cm), and − 2.7 cm (± 0.7 cm) in the multi-slice sequences. There was a statistically significant difference between the single-slice and multi-slice sequences in the cervix position (*p* = 0.003), but not for the position of the bladder (*p* = 0.322) and anorectal junction (*p* = 0.465) (Fig. 2).

At straining, the mean positions of bladder, cervix, and anorectal junction were − 1.1 cm (± 1.1 cm), − 3.2 cm (± 1.1 cm), and − 0.7 cm (± 1.4 cm) in the single-slice sequences. Same organ positions were − 1.5 cm (± 1.0 cm), − 3.4 cm (± 1.1 cm), and −1.0 cm (± 1.3 cm) in the multi-slice sequences. There was a statistically significant difference between the single-slice and multi-slice sequences in the bladder position (*p* = 0.019), but not for the position of the cervix (*p* = 0.223) and anorectal junction (*p* = 0.158) (Fig. 2).

At rest, the mean difference (and range) between measurements in the single- and multi-slice sequence was − 0.1 cm (− 0.8 to + 0.2 cm) for the bladder, − 0.4 cm (− 1.4 to + 0.4 cm) for the cervix, and − 0.1 cm (− 0.7 to + 0.7 cm) for the anorectal junction. At straining, the mean differences were + 0.4 cm (− 0.3 to + 2.5 cm) for the bladder, + 0.2 cm (− 0.9 to + 1.8 cm) for the cervix, and + 0.3 cm (− 0.7 to + 2.5 cm) for the anorectal junction. All but one measurement in each compartment were inside the 95% confidence intervals (CI).

#### Pelvic organ point measurements in symptomatic patients

**Fig. 3:**
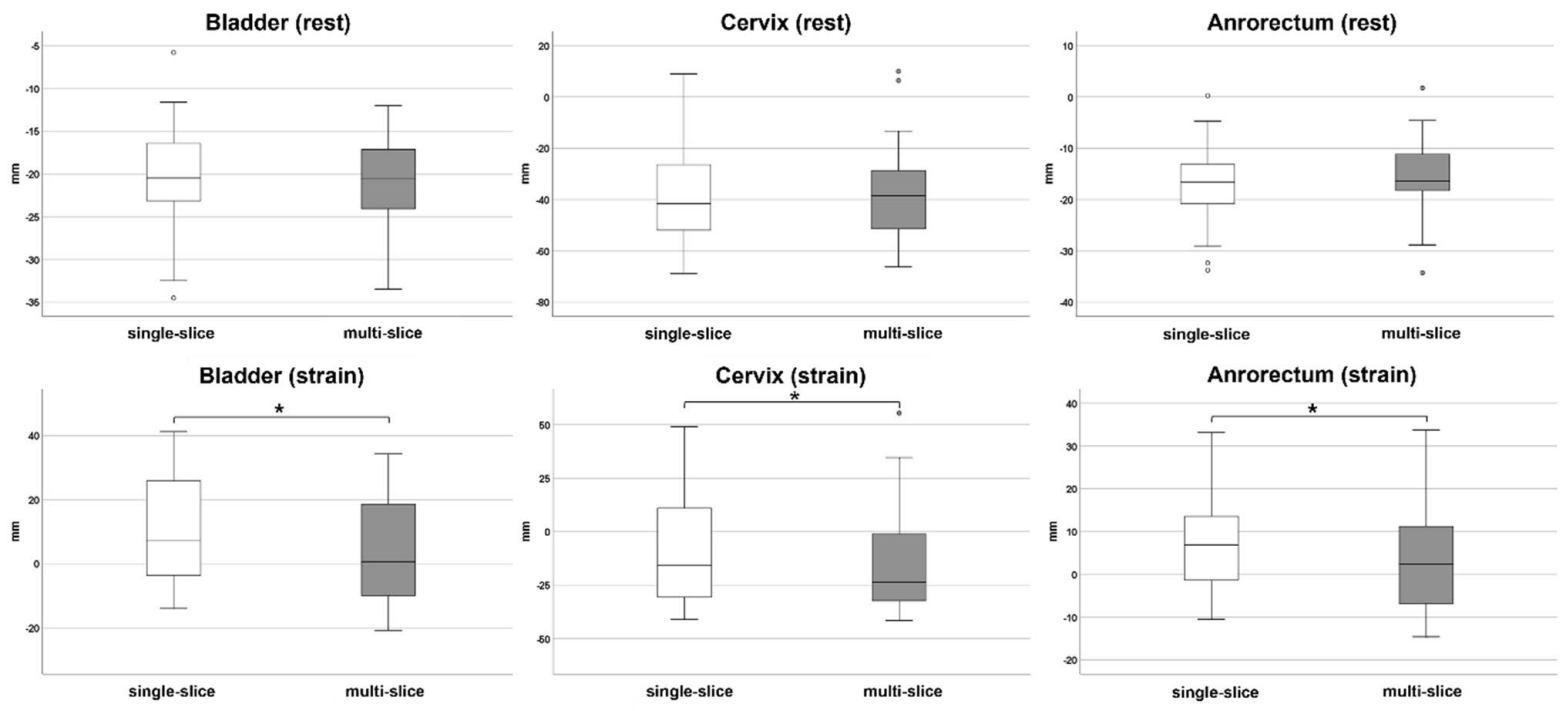
Box plots comparing organ positions in the *y*-axis at rest (top row) and straining (bottom row) between single-slice (white box) and multi-slice sequences (grey box) in symptomatic patients. Asterisks indicate statistical significance

At rest, the mean positions of bladder, cervix, and anorectal junction were − 2.0 cm (± 0.6 cm), − 3.8 cm (± 2.0 cm), and − 1.7 cm (± 0.8 cm) in the single-slice sequences. Same organ positions were − 2.1 cm (± 0.6 cm), − 3.7 cm (± 2.0 cm), and − 1.6 cm (± 0.9 cm) in the multi-slice sequences. There was no statistically significant difference between the single-slice and multi-slice sequences (bladder: *p* = 0.927, cervix: *p* = 0.274, anorectal junction: *p* = 0.280) (Fig. 3).

At straining, the mean positions of bladder, cervix, and anorectal junction were + 1.1 cm (± 1.8 cm), − 0.7 cm (± 2.9 cm), and + 0.7 cm (± 1.3 cm) in the single-slice sequences. Same organ positions were + 0.4 mm (± 1.7 cm), − 1.4 cm (± 2.6 cm), and + 0.4 cm (± 1.3 cm) in the multi-slice sequences. There was a statistically significant difference between the single-slice and multi-slice sequences for all three organ points (bladder: *p* < 0.001, cervix: *p* = 0.011, anorectal junction: *p* = 0.042) (Fig. 3).

**Fig. 4:**
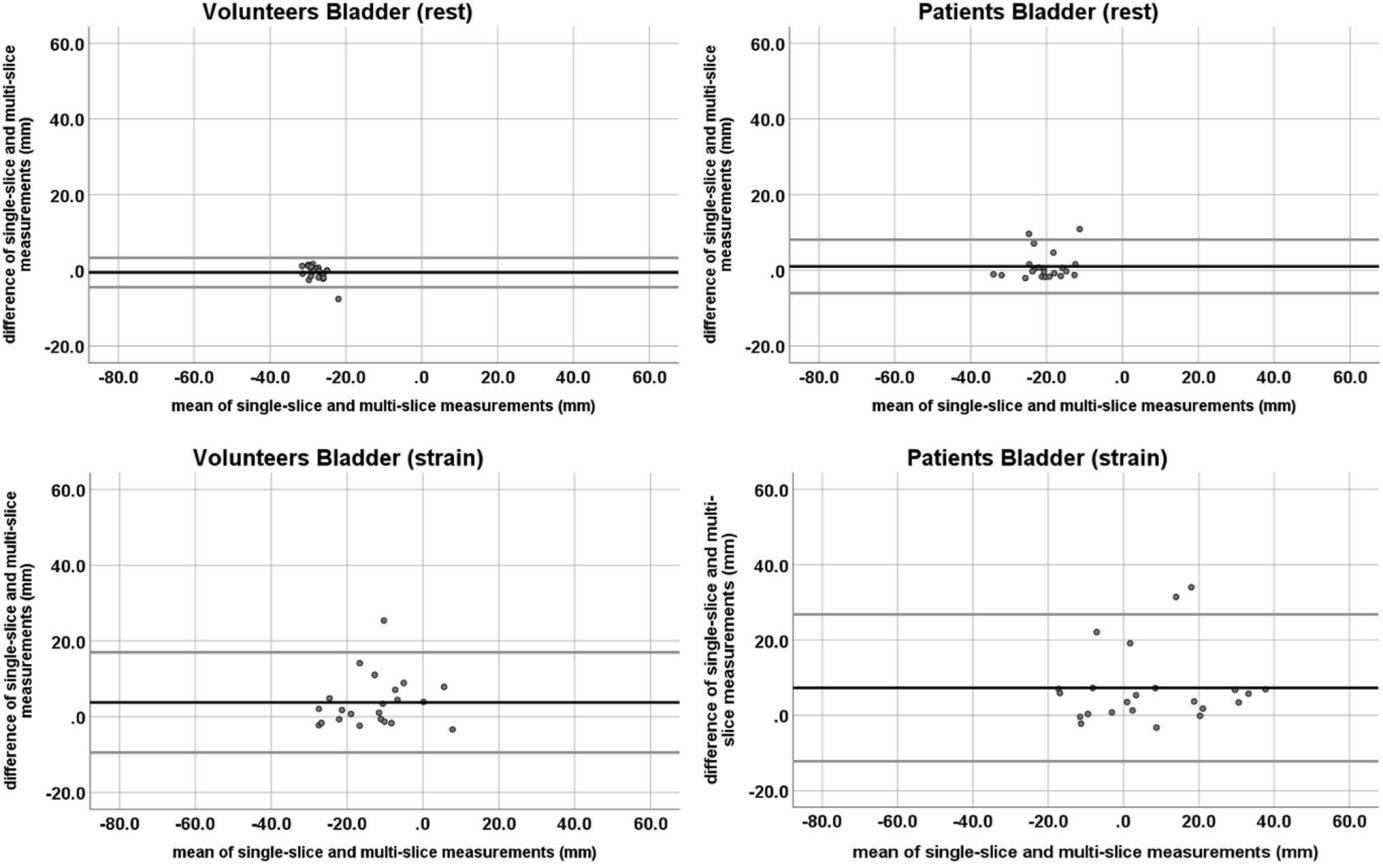
Bland–Altman plots of the bladder organ point measurements on single-slice and multi-slice sequences in volunteers (left row) and patients (right row) at rest (top row) and straining (bottom row). The black line depicts the mean difference. The top and bottom grey lines denote the upper and lower limits of agreement

**Fig. 5:**
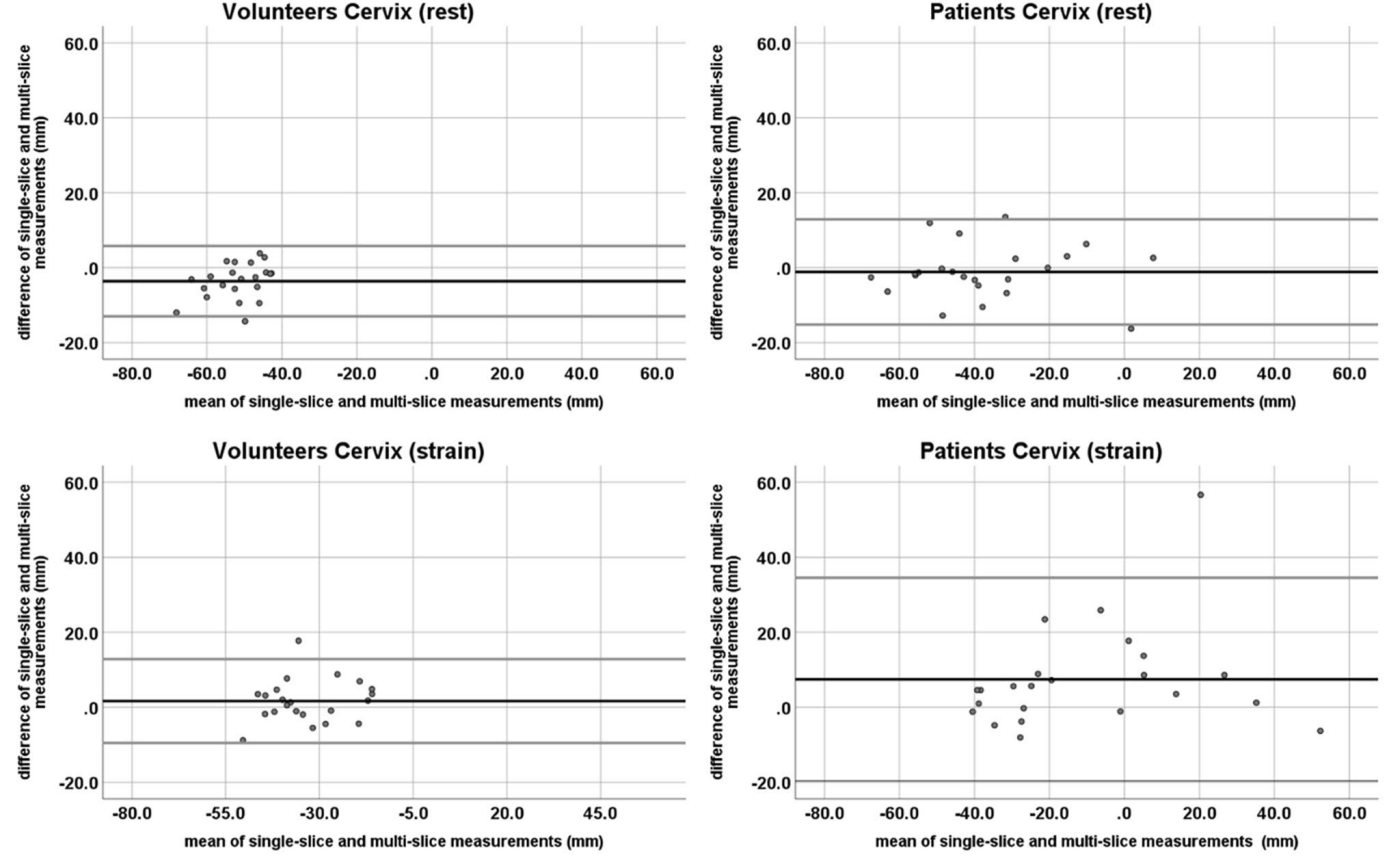
Bland–Altman plots of the cervix organ point measurements on single-slice and multi-slice sequences in volunteers (left row) and patients (right row) at rest (top row) and straining (bottom row). The black line depicts the mean difference. The top and bottom grey lines denote the upper and lower limits of agreement

**Fig. 6:**
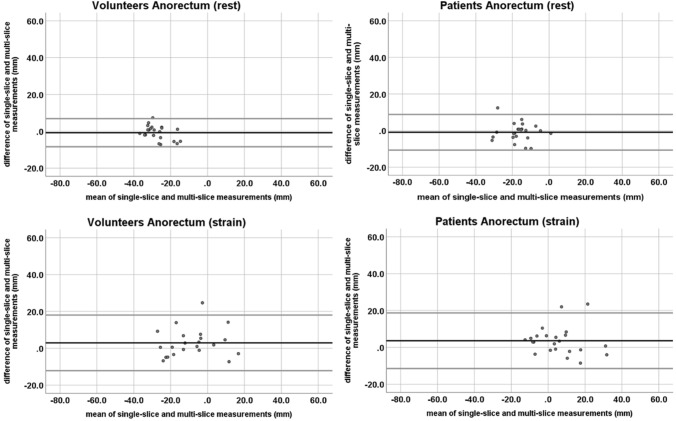
Bland–Altman plots of the anorectal joint measurements on single-slice and multi-slice sequences in volunteers (left row) and patients (right row) at rest (top row) and straining (bottom row). The black line depicts the mean difference. The top and bottom grey lines denote the upper and lower limits of agreement

At rest, the mean difference (and range) between measurements in the single- and multi-slice sequence was 0.1 cm (− 0.2 to + 1.1 cm) for the bladder, − 0.1 cm (− 1.6 to + 1.4 cm) for the cervix, and 0.0 cm (− 1.0 to + 1.2 cm) for the anorectal junction. At straining, the mean difference was + 0.7 cm (− 0.3 to + 3.4 cm) for the bladder, + 0.7 cm (− 0.8 to + 5.7 cm) for the cervix, and + 0.4 cm (− 0.9 to + 2.4 cm) for the anorectal junction. In each compartment, there were one to two measurements outside of the 95% CI (Figs. 4, 5, 6).

### Interreader agreement

For the single-slice sequences at rest and straining, the interobserver agreement was excellent for the bladder (ICC 0.989, 95% CI 0.971–0.995) and cervix (ICC 0.953, 95% CI 0.880–0.981) and good for the anorectal junction (ICC 0.679, 95% CI 0.189–0.873).

For the multi-slice sequences at rest and straining, the interobserver agreement was excellent for the bladder (ICC 0.968, 95% CI 0.918–0.987), cervix (ICC 0.917, 95% CI 0.790–0.967), and anorectal junction (ICC 0.872, 95% CI 0.676–0.949).

### Pelvic organ prolapse grading

#### Asymptomatic volunteers

Three cases (13.6%) had a grade 1 descent of the anterior compartment on the single-slice sequence, of which two were also evident on the multi-slice sequence and one was not. In that case, the bladder point was measured at 1.5 cm below the PCL in the single-slice sequence but only at 1.0 cm below PCL in the multi-slice sequence (difference of 0.5 cm), hence, the formal threshold of 1.0 cm for calling a grade 1 descent was not reached on the multi-slice sequence (Table 2).

**Table 2:** Cases with discrepancy of POP grading between measurements on single-slice and multi-slice sequences

	Anterior compartment	Middle compartment	Posterior compartment	Straining effort	Measurement difference (cm)
Volunteer				Good	0.5
Single-slice	Grade 1				
Multi-slice	None				
Patient				Good	1.1
Single-slice	Grade 1				
Multi-slice	None				
Patient				Good	0.4
Single-slice	Grade 1				
Multi-slice	None				
Patient				Good	0.1
Single-slice	Grade 2				
Multi-slice	Grade 1				
Patient				Suboptimal	2.1/2.2
Single-slice	Grade 1		Grade 1		
Multi-slice	None		None		
Patient				Suboptimal	3.1
Single-slice	Grade 2				
Multi-slice	None				
Patient				Good	0.6/0.5
Single-slice	Grade 2		Grade 1		
Multi-slice	Grade 1		None		
Patient				Good	1.5
Single-slice		Grade 1			
Multi-slice		None			
Patient				None	2.8/3.9
Single-slice	Grade 2		Grade 2		
Multi-slice	None		None		

No descent of the middle compartment was seen.

Two cases (9.0%) had a grade 1 descent of the posterior compartment on single-slice sequence, which was also evident on the multi-slice sequence.

#### Symptomatic patients

Fourteen symptomatic patients (60.8%) presented with a descent of the anterior compartment (grade 1: *n* = 6, grade 2: *n* = 8) on the single-slice sequence. In 7 of these 14 cases (50%), the multi-slice sequence did not depict a descent (*n* = 5) or only depicted a grade 1 instead of grade 2 descent (*n* = 2). Of those cases that were not depicted in the multi-slice sequence, three cases had a grade 1 descent and two cases a grade 2 descent. Three of those five missed cases had either a suboptimal (*n* = 2) or no visible (*n* = 1) straining effort in the multi-slice sequence. In those two cases where a grade 1 instead of grade 2 descent was depicted on the multi-slice sequence, the straining effort was scored as good and the difference in measurements between the single- and multi-slice sequence was 0.1 and 0.6 cm, respectively (Table 2).

Four symptomatic patients (17.3%) presented with a descent of the middle compartment (grade 1: *n* = 3, grade 2: *n* = 1) on the single-slice sequence. One of these cases with grade 1 descent was not captured on the multi-slice sequence despite a subjectively good straining effort (the difference in measurement between the single- and multi-slice sequence was 1.5 cm) (Table 2).

Seven symptomatic patients (30.4%) had a descent of the posterior compartment (grade 1: *n* = 6, grade 2: *n* = 1) on the single-slice sequence. The multi-slice sequence did not capture a descent in 3 of those cases. These included two cases with a grade 1 descent (one with suboptimal straining effort, the other with a good straining effort, but did not meet the threshold for a grade 1 descent with a difference of 0.5 cm between the single- and multi-slice sequence) and one case with a grade 2 descent, where there was no visible straining effort in the multi-slice sequence (Table 2).

**Fig. 7:**
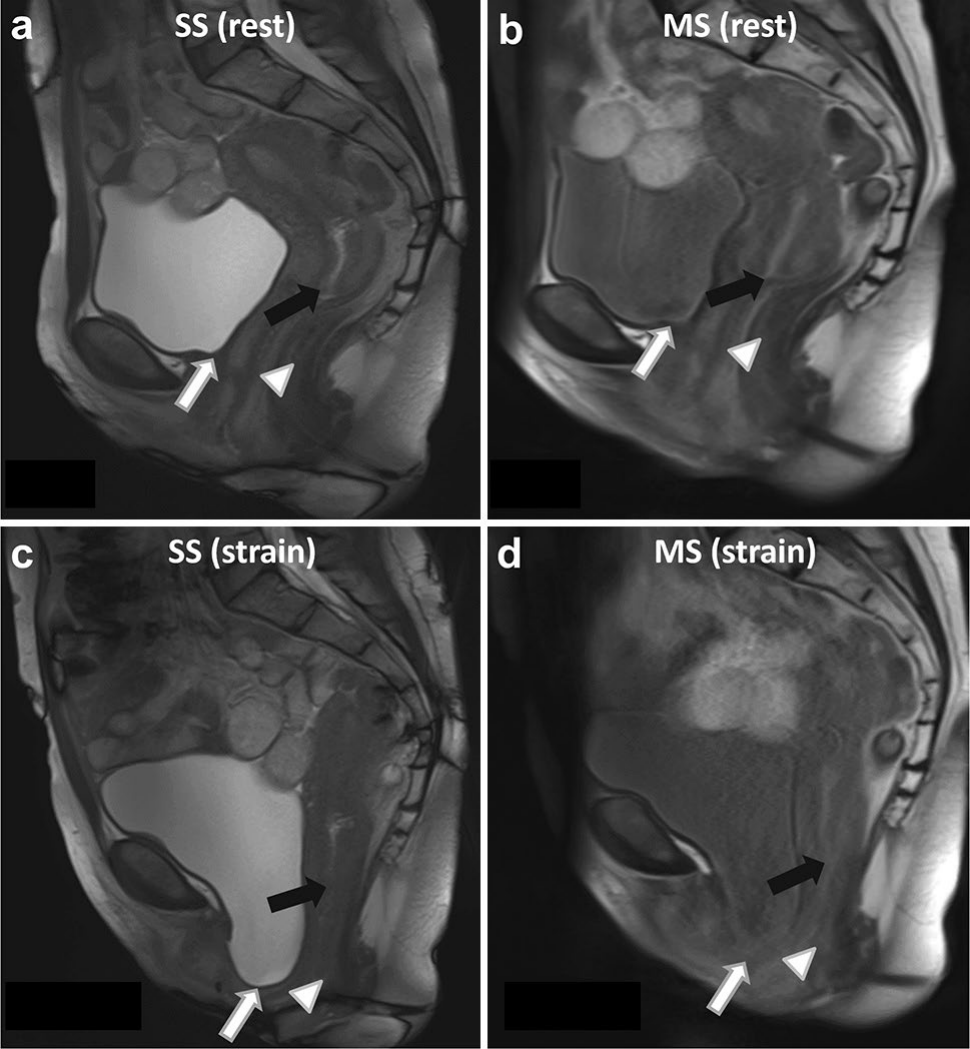
38-year old primiparous woman with symptomatic pelvic organ prolapse in involving all three compartments and a POP-Q score of III. Sagittal single-slice (**a**, **c**) and multi-slice (**b**, **d**) MR images at rest (**a**, **b**) and maximum straining (**c**, **d**) are shown. The bladder (white arrow), cervix (black arrow) and anorectal junction (white arrowhead) are annotated. At rest, the bladder point was 1.6 cm and 1.6 cm, the cervix point was 4.1 cm and 3.7 cm, and the anorectal junction was 1.4 cm and 1.0 cm above the PICS line in the single-slice and multi-slice sequences, respectively. During maximum straining, the bladder point was 4.1 cm and 3.4 cm below, the cervix point was 0.2 cm and 0.0 cm above, and the anorectal junction was 3.1 cm and 3.1 cm below the PICS line in the single-slice and multi-slice sequence, respectively. Differences between single and multi-slice sequences were small (0.1–0.4 cm at rest and 0.0–0.7 cm at straining) and there was no difference in the POP grading between the single-slice and multi-slice sequence (grade 2 descent of the anterior compartment, and grade 1 descent of the posterior compartment)

Figure 7 shows an example of single-slice and multi-slice sequence images from a symptomatic patient with POP.

## Discussion

In this prospective feasibility study, we explored whether dynamic MR multi-slice sequences of the pelvis can be used for the assessment of POP and compared it to the reference standard of midsagittal single-slice sequences. We found that dynamic evaluation of the pelvic organs is feasible on multi-slice sequences and shows excellent interreader agreement.

While the multi-slice sequences consistently displayed the three examined organ points, the single-slice sequences failed to fully visualize the cervix point in one-third of the cases. Since the single-slice sequence is obtained in the midsagittal plane, there is a potential that structures located slightly off the midline may not be entirely visible.

Moreover, our analysis revealed that single-slice and multi-slice sequences were comparable for images captured during rest. Notably, when the organ points were entirely visible in the single-slice sequence, no statistically significant variance in organ point measurements was observed between the two imaging modalities. Conversely, the differences in organ point measurements between single- and multi-slice sequences increased in the straining images and became statistically significant for all three compartments in patients.

In MR examinations of the pelvic floor, where patients are scanned in the supine position, the impact of gravity is disregarded, and the risk of inaccurate staging of POP may arise if patients fail to perform the straining maneuver appropriately [18, 19]. In our cohort of premenopausal symptomatic patients, anterior compartment prolapse (i.e., cystocele) was more prevalent than prolapse of the middle and posterior compartments (60.8% vs. 17.3% and 30.4%), which is also consistent with the experience in clinical practice [20]. This is believed to be linked to the complexity of the underlying support structures for the anterior compartment where defects in each of these components can lead to development of prolapse [21]. Presence of higher-grade anterior compartment prolapse could possibly affect the depiction of middle and posterior compartment prolapse when imaged in the supine position, although the extent to which examination in supine position could obscure or underestimate middle and posterior compartment prolapse remains to be elucidated in future studies [19]. The severity of POP is also influenced by age, with postmenopausal women showing increased pelvic organ movement [22, 23]. As this study only included premenopausal women, the benefit of dynamic multi-slice sequences may have been underestimated. Therefore, future studies will need to investigate the use of dynamic multislice sequences in postmenopausal women as well.

An adequate straining maneuver where the patient is bearing down as much as possible is an essential prerequisite for a good dynamic pelvic MRI exam. Insufficient straining at the time of image acquisition had a negative impact on the measurement accuracy on the multi-slice sequence. In fact, we found that there was a statistically significant higher proportion of cases with insufficient or no straining effort on the multi-slice sequence compared to the single-slice sequence. The observed variation may be attributed to the mode of image acquisition, whereby three consecutive straining maneuvers were performed for the single-slice sequences, in contrast to one maneuver for the multi-slice sequence. In a previous study that investigated the reproducibility of straining and prolapse, it was demonstrated that the prolapse size could significantly increase from the first to the third Valsalva attempt [18].

For the multi-slice sequences, symptomatic patients and healthy volunteers were instructed to perform a single maximum straining maneuver, image acquisition was timed to capture this instant of maximum bearing down, and there is the possibility that the acquired images in multi-slice sequences did not always coincide perfectly in time with the instant of maximum straining.

Furthermore, it could have been challenging for the participants to maintain a consistent straining maneuver throughout the entire 20 s image acquisition period. These constraints could be addressed in future studies by acquiring three consecutive straining maneuvers with repeated acquisition of the multi-slice sequence. Moreover, employing new MRI acceleration techniques that utilize deep learning AI-based image reconstruction to reduce the acquisition time to less than 20 s would be advantageous [24, 25].

Although there were statistically significant differences between the single-slice and multi-slice sequences, the mean differences in absolute numbers were small (all less than 1.0 cm). We thus investigated whether the use of multi-slice sequences affected POP grading. In symptomatic patients, a higher-grade POP (grade 2) was missed in only two cases with the multi-slice sequence (both cases with suboptimal or no straining effort) and underestimated in another two patients (in which the respective threshold was not met). In those latter two cases the measurement differences between the multi-slice and single-slice sequence were only 0.1 cm und 0.6 cm. Lower grade POP was missed in three symptomatic patients with the multi-slice sequence, one of those due to suboptimal straining effort. In volunteers, the POP grading was similar between single-slice and multi-slice sequence.

We also noted a greater variability in measurements between single- and multi-slice sequences in symptomatic patients with POP than in asymptomatic volunteers. We postulate that this variance may be attributed to technical and patient-specific aspects of the dynamic image acquisition process. In patients experiencing POP symptoms, there is a higher degree of organ mobility during straining due to the increased structural laxity, resulting in a broader range of downward mobility during straining. Conversely, organ mobility is lower in asymptomatic nulliparous women [26]. Especially in symptomatic patients the timing of image acquisition to capture the moment of maximum straining appears crucial and the greater measurement variability is therefore probably also related to suboptimal timing of image acquisition with the straining maneuver.

Contemporary management of pelvic floor disorders involves not only the quantification of POP through measurement of organ points using reference lines but also more tailored and defect-specific treatment approaches that require imaging to deliver more detailed information on structural defects of the pelvic floor [27]. This customized treatment approach requires an improved understanding of the pathogenesis of POP through a sophisticated assessment of pelvic floor support structures. The evaluation of the pelvic floor muscles and ligaments is becoming increasingly important in management of pelvic floor disorders. These supporting structures are not exclusively located in the midline but rather span the pelvis in a three-dimensional manner, each with its distinct origin-insertion pairs. For example, the levator ani muscle has different anterior and posterior subdivisions, each with its own unique mechanical effects related to different pelvic functions not all fully elucidated yet [28]. Several prior studies were able to show that MRI can accurately depict the anatomy of these levator ani subdivisions on static images [28–30]. While dynamic evaluation of the levator ani subdivisions can be accomplished through transperineal or translabial ultrasound, MRI-based dynamic assessment of these structures has not been explored. This is mainly due to the current clinical practice of using single-slice sequences in dynamic MRI of the pelvic floor, which limits the assessment of structures that are not situated in the midline of the pelvis. Dynamic multi-slice sequences of the pelvic floor hold great promise for future applications, as they could enable the study of changes in the movement vectors of organs and pelvic floor structures in all three planes. This would enhance our understanding of the complex three-dimensional interplay between different pelvic floor structures in both asymptomatic and symptomatic women with pelvic floor disorders.

It should be noted that dynamic images at rest and maximum straining were used in this study. However, previous research has shown that performing MR defecography by instilling rectal gel and incorporating a defecation phase enhances the visibility of pelvic organ prolapse and reveals other underlying conditions (e.g., intussusception, rectoceles, enteroceles) that were not initially suspected clinically or detected through MRI imaging during straining alone [31–35]. Therefore, current joint multispecialty recommendations advise using MR defecography for the evaluation of pelvic floor disorders. Furthermore, MR defecography is considered superior to straining MRI alone for this purpose according to the American College of Radiology (ACR) appropriateness criteria [36]. In addition, the Valsalva maneuver (for straining MRI) and defecation phase (for MR defecography) should be repeated several times to ensure that maximum prolapse is depicted [15, 18, 37]. Therefore, it is advisable to include MR defecography into every MRI protocol for the evaluation of pelvic floor disorders, this could also be considered for those patients presenting with anterior and middle compartment-predominant symptoms as additional unsuspected pathologies could be unmasked. In this setting, dynamic multi-slice sequences could be integrated as a supplementary technique to the current MRI imaging protocol (including dynamic single-slice MR defecography) depicting structures of the pelvic floor that are located off the midline, such as muscles and ligaments.

Our study has several limitations. First, our sample size was relatively small. However, considering the exploratory nature of our study and our aim to evaluate feasibility, we believe that the sample size was adequate for our objectives. Second, the multi-slice sequences only included one straining maneuver as opposed to the three straining maneuvers for the single-slice sequence. This could have influenced the results as it has been shown that there can be significant variations in POP grade between consecutive straining maneuvers [18]. It is recommended to use more than one straining maneuver for future applications of dynamic multi-slice sequences. In addition, the multi-slice sequences were acquired after the single-slice sequences and a potential negative impact on straining performance due to fatigue cannot be ruled out. Thirdly, while the interobserver agreement was good to excellent, it is important to note that the varying levels of experience among the readers and the fact that the second reader assessed a random subset of 20 cases, rather than the entire cohort, present some limitations. Despite these factors, our results indicate that the measurements are reproducible even among readers with differing levels of expertise. Fourth, our study did not include MR defecography and no defecation phase was performed. Performing a defecation phase induces the maximum stress to the pelvic floor and it has been shown that MR defecography increased conspicuity of pelvic organ prolapse in all three compartments, unmasks unsuspected pathologies and improves detection of posterior compartment abnormalities (e.g., rectocele, intussusception) [32–35, 37–39].

Last, we only compared differences of organ points in the *y*-axis (i.e., craniocaudal direction) between the two cohorts and did not analyze differences of coordinates in the *x*- (antero-posterior) or *z*-axis (medio-lateral), which should be the objective of future studies.

## Conclusion

Dynamic multi-slice sequences are feasible and could be incorporated into existing imaging protocols as an adjunct tool for the evaluation of pelvic floor disorders. Multi-slice sequences of the pelvis increase the visibility of organ points compared to single-slice sequences at rest and straining and can depict pelvic organ prolapse in images acquired with sufficient straining effort. Further work is needed to optimize the depiction of the maximum straining effort with multi-slice sequences.

## Supplementary Information

Below is the link to the electronic supplementary material.

Supplementary file1 (TIF 5889 kb)—**Supplementary Figure 1** Single-slice (a) and multi-slice (b, c) sequences at rest. In the midsagittal single-slice sequence (a) the bladder point (white arrow) and anorectal junction (white arrowhead) are visualized whereas the cervix is not. The uterus is only partly visible (black arrow in a). In the corresponding multi-slice sequences (b, c) all organ points can be visualized, the bladder point (white arrow in b) and anorectal junction (white arrowhead in b) in the midline and the cervix (black arrow in c) located two slices off the midline.

Supplementary file2 (DOCX 13 kb)
