# Supplementary figures and images for: Pelvic organ movements in asymptomatic nulliparous and symptomatic premenopausal women with pelvic organ prolapse in dynamic MRI: a feasibility study comparing midsagittal single-slice with multi-slice sequences
Source: Abdom Radiol (NY). 2023 May 19;48(8):2658–71. doi: 10.1007/s00261-023-03944-8 (PMC10333376; doi:10.1007/s00261-023-03944-8)

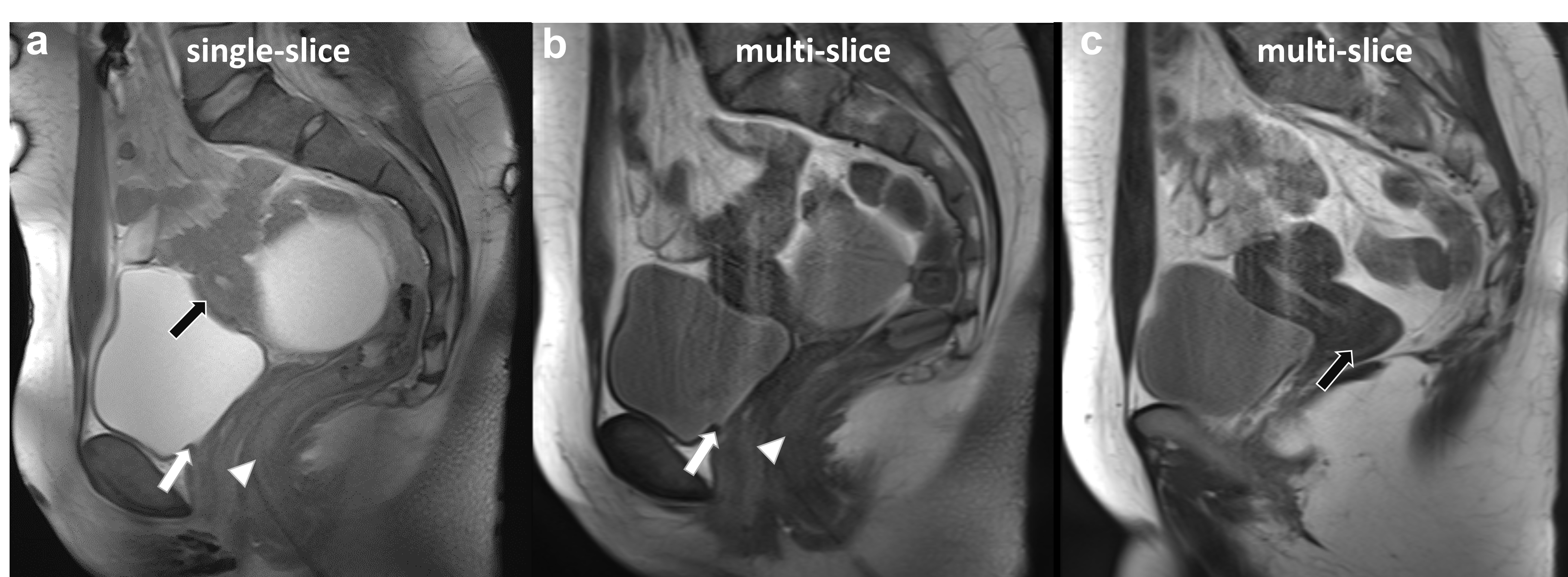

Supplement: Supplementary file 1 — Supplementary file1 (TIF 5889 kb)—Supplementary Figure 1 Single-slice (a) and multi-slice (b, c) sequences at rest. In the midsagittal single-slice sequence (a) the bladder point (white arrow) and anorectal junction (white arrowhead) are visualized whereas the cervix is not. The uterus is only partly visible (black arrow in a). In the corresponding multi-slice sequences (b, c) all organ points can be visualized, the bladder point (white arrow in b) and anorectal junction (white arrowhead in b) in the midline and the cervix (black arrow in c) located two slices off the midline. [file 261_2023_3944_MOESM1_ESM.tif]
