# Supplementary material for: Pelvic organ movements in asymptomatic nulliparous and symptomatic premenopausal women with pelvic organ prolapse in dynamic MRI: a feasibility study comparing midsagittal single-slice with multi-slice sequences
Source: Abdom Radiol (NY). 2023 May 19;48(8):2658–71. doi: 10.1007/s00261-023-03944-8 (PMC10333376; doi:10.1007/s00261-023-03944-8)
Supplement: Supplementary file 2 — Supplementary file2 (DOCX 13 kb) [file 261_2023_3944_MOESM2_ESM.docx]

Supplementary Table 1: Grading of pelvic organ prolapse

|  | **Anterior Compartment** | **Middle Compartment** | **Posterior Compartment** |
| --- | --- | --- | --- |
| **Normal** | up to 1 cm below PCL | up to 1 cm below PCL | up to 3 cm below PCL |
| **Grade 1** | 1 – 3 cm below PCL | 1 - 3 cm below PCL | 3 – 5 cm below PCL |
| **Grade 2** | 3 – 6 cm below PCL | 3 – 6 cm below PCL | > 5 cm below PCL |
| **Grade 3** | > 6 cm below PCL | > 6 cm below PCL | - |
| * based on consensus recommendations [15]  PCL = pubococcygeal line | | | |
